# Deferred

**Published:** 1885-07

**Authors:** 


### DEFERRED.

Considerable matter that had been prepared for this number is of necessity left over until next month. The continuation of the report of the meeting of The Dental Society of the State of New York, and the book notices that are due, form a part of it. Both shall have space in the next number.

